# Ceuta 2026: the case for guaranteed health surge capacity at Europe’s external borders

**DOI:** 10.1093/eurpub/ckag182

**Published:** 2026-10-09

**Authors:** Zeus Aranda

**Affiliations:** Department of Public Health and Maternal-Infant Health, School of Medicine, Complutense University of Madrid, Madrid, España

On 30–31 July 2026, tens of thousands of people crossed from Morocco into the Spanish exclave of Ceuta in under 48 hours: the largest single migratory surge recorded at a European external border. Spain’s government confirmed 72 000 arrivals in a statement of 10 August, an episode that left 82 dead, mainly by drowning, according to official Spanish records, and a further 67 estimated on the Moroccan side by the Spanish NGO Caminando Fronteras, per figures endorsed by Human Rights Watch as of 21 August [1]. Some 5000 people who had crossed in late July remained in Ceuta by 13 August, per the interior ministry, a figure local organizations put as high as 10 000 [1]. According to the health minister, in her appearance before the Spanish Parliament on 27 August, health services recorded 1146 attendances on 30 July alone and between 250 and 350 a day thereafter, with some 75 more emergency presentations daily than a year earlier and a peak of 35 migrant inpatients on 13 August, in a system serving 84 000 residents [2]. Ceuta illustrates a recurring pattern: sudden mass arrivals overwhelm local health and reception systems, harming migrants and host communities alike, before any coordinated European public health response can be mobilized.

This is not new. On 17–18 May 2021, an estimated 10 000–12 000 people crossed the same border and around 1500 were unaccompanied minors, per official figures; reception centres and children’s shelters were overwhelmed [3]. On 12–13 September 2023, Italian authorities estimated 7000 arrivals on Lampedusa, whose resident population is under 6000, again exceeding capacity many times over [4]. In neither case is there concrete evidence of migrants’ health problems, beyond initial hypothermia, fatigue and Red Cross statements on precarious living conditions, difficulties in meeting basic needs, the absence of health protocols and overstretched operations [3, 4]. What had been documented, before that episode, were weaknesses in preparedness: in a survey of clinicians who staffed the Lampedusa hotspot clinic between 2020 and 2022, reported previously in this journal, 93% had begun work without training and 80% had never had access to protocols or guidelines [5]. Each episode produced emergency national measures, political controversy over border security and burden-sharing, and institutional silence until the next surge exposed the same structural gaps.

Ceuta in 2026 differs from these earlier episodes in its scale and its explicit health dimension. On 3 August, the Ceuta councillor responsible for child protection put occupancy of the city’s facilities for unaccompanied minors at 2865% [6]. The public health coordination committee created for the crisis reported, on 14 September, six confirmed tuberculosis cases since 30 July, with no evidence of clustering [7], and 15 cases of varicella, 11 of them confirmed and none with complications [8]. On 27 August, the health minister told the Spanish Parliament of some 60 cases of gastroenteritis caused by enteroinvasive *Escherichia coli*, which she attributed to unsafe drinking water [2].

Yet the visible response of the European Union (EU) was organized almost entirely around border security and inter-governmental politics rather than health. The European Commission offered to deploy Frontex, which Spain initially declined; while the Commission president called for solidarity, some EU leaders linked the crisis to Spain’s migration policy, and Italy reinstated Schengen controls on Spain travellers [9]. Health institutions left no comparable trace: no statement, deployment or coordinating role has been publicly reported for the European Centre for Disease Prevention and Control (ECDC), the Commission’s health directorate or the Union Civil Protection Mechanism (UCPM), nor for WHO/Europe, which sits outside the EU framework. Whether quieter technical exchanges took place is not on the public record.

Two EU instruments were, in principle, available. The first, surveillance and coordination architecture, is Regulation (EU) 2022/2371 [10], providing early-warning notification and Health Security Committee coordination, and giving ECDC a standing role in surveillance and risk assessment. Under Article 19 of the Regulation, an alert must be notified where a threat is unusual or severe, affects or may affect more than one Member State, and requires or may require a coordinated response. Whether Ceuta’s gastroenteritis, varicella and tuberculosis met this threshold cannot be established with confidence from the available information, and whether an alert was notified through the Early Warning and Response System is not publicly known. Framing them as a threat from the migrant population, moreover, risks a ‘double stigma’ (social exclusion reinforced by fear of infection) [11], when their documented drivers are overcrowding, poor sanitation and interrupted care, not the migrants themselves. Even if met, the Regulation’s tools stop short of deploying medical teams or supplies; that capacity lies with the UCPM, through which the European Medical Corps and rescEU stockpiles can do so [12]. Neither instrument is publicly recorded as having been used for Ceuta.

Part of the problem is structural: the EU Pact on Migration and Asylum’s operative rules, including a mandatory screening and health assessment, entered into application weeks earlier, in June 2026 [13]. The Ceuta surge was the new framework’s first stress test. The Pact does anticipate exceptional migratory pressure: Regulation (EU) 2024/1359 on situations of crisis and force majeure lets a Member State facing mass arrivals extend registration and procedural deadlines and request relocation or other solidarity support [14]. But none of these arrangements guarantee timely health surge capacity: they extend legal and administrative deadlines, not hospital beds, clean water, sanitation or trained staff, which is precisely where Ceuta’s response was least prepared. The UCPM and its European Medical Corps exist to mobilize emergency medical capacity, but they are generalist instruments for discrete disasters [12], not recurring sudden mass arrivals at external borders. No Spanish request for UCPM assistance has been made public, and the mechanism requires none: activation turns on a national decision, leaving deployment ad hoc rather than guaranteed. What is missing is not capacity, but a mechanism that is health-specific, automatically triggered, and decoupled from the political discretion that now determines whether, and how fast, instruments like the UCPM are invoked.

Strengthening the European response need not require a wholly new architecture, nor centralizing migration policy, on which member states remain deeply divided. It requires adapting the logic of the UCPM, its European Medical Corps and rescEU stockpiles to border health emergencies, through a dedicated mechanism: mobile clinical and mental health teams, pre-positioned medical supplies and dedicated funding, drawing on standing support from ECDC for epidemiological risk assessment and outbreak-assistance teams, and from WHO/Europe for technical guidance and cross-border coordination with Morocco and neighbouring states.

The trigger would combine an absolute threshold, such as arrivals exceeding a set number within 48–72 hours, with a relative one measured against local reception capacity or population, so Lampedusa’s 6000 residents and Ceuta’s 84 000 are not measured by the same yardstick. The Commission, not the Member State, would certify the threshold was met, drawing on data from Frontex and the EU Agency for Asylum (EUAA). What would follow automatically is the offer, not the deployment: certification would trigger assessment, mobilization of teams and stockpiles, and a formal offer of assistance within a fixed period, while entry and integration into the national response would remain subject to host-state consent. Member State competence over the organization of health services would thus be preserved, and what is removed is only the discretion that now determines whether assistance is offered at all, and how fast. EU4Health is one plausible route for financing EU-level teams and stockpiles, though it would require an appropriate legal and budgetary basis; on the rescEU model, such capacities are funded by the Union rather than the host state, whose role would be to integrate them locally.

None of this requires new institutions: a data-based trigger at the Commission, informed by Frontex and EUAA reporting; a border-health module added to rescEU’s capacity list; and a reformed ECDC mandate alongside a formal WHO/Europe agreement. Such a mechanism would protect the health not only of people arriving, but also of host communities and of the healthcare workers currently absorbing these shocks.

Ceuta in 2026, like in 2021 and Lampedusa in 2023, shows that mass-arrival border crises are a recurring feature of Europe’s frontier, with costs falling disproportionately on a handful of small, peripheral municipalities. Until the EU treats these episodes as the public health emergencies they demonstrably are, the next sudden crossing will again find health systems at Europe’s edge facing it alone. As argued previously in this journal, when the mechanisms that safeguard collective health, including humanitarian access, are eroded by unilateral action, it is essential to counter that trend [15]. The proposal presented here, in which European public health defends universalism at its external borders, sets an example of institutional solidarity grounded in universality, reciprocity and dignity rather than contingent preferences.

## Data Availability

All sources on which this commentary is based are publicly available.
